# A Three-Arm Randomized Controlled Trial Using Ecological Momentary Intervention, Community Health Workers, and Video Feedback at Family Meals to Improve Child Cardiovascular Health: The Family Matters Study Design

**DOI:** 10.21203/rs.3.rs-2662682/v1

**Published:** 2023-03-22

**Authors:** Jerica M. Berge, Amanda C. Trofholz, Marah Aqeel, Kristin Norderud, Allan Tate, Angela R. Fertig, Katie Loth, Tai Mendenhall, Dianne Neumark-Sztainer

**Affiliations:** University of Minnesota; University of Minnesota; University of Minnesota; University of Minnesota; University of Georgia; University of Minnesota; University of Minnesota; University of Minnesota; University of Minnesota

**Keywords:** Child Cardiovascular Health, Mixed-Methods Randomized Controlled Trial, Ecological Momentary Intervention, Community Health Workers, Video Feedback, Virtual

## Abstract

**Background:**

Numerous observational studies show associations between family meal frequency and markers of child cardiovascular health including healthful diet quality and lower weight status. Some studies also show the “quality” of family meals, including dietary quality of the food served and the interpersonal atmosphere during meals, is associated with markers of child cardiovascular health. Additionally, prior intervention research indicates that immediate feedback on health behaviors (e.g., ecological momentary intervention (EMI), video feedback) increases the likelihood of behavior change. However, limited studies have tested the combination of these components in a rigorous clinical trial. The main aim of this paper is to describe the *Family Matters* study design, data collection protocols, measures, intervention components, process evaluation, and analysis plan.

**Methods/design:**

The *Family Matters* intervention utilizes state-of-the-art intervention methods including EMI, video feedback, and home visiting by Community Health Workers (CHWs) to examine whether increasing the quantity (i.e., frequency) and quality of family meals (i.e., diet quality, interpersonal atmosphere) improves child cardiovascular health. *Family Matters* is an individual randomized controlled trial that tests combinations of the above factors across three study Arms: (1) EMI; (2) EMI+Virtual Home Visiting with CHW+Video Feedback; and (3) EMI+Hybrid Home Visiting with CHW+Video Feedback. The intervention will be carried out across 6 months with children ages 5-10 (n=525) with increased risk for cardiovascular disease (i.e., BMI ≥75%ile) from low income and racially/ethnically diverse households and their families. Data collection will occur at baseline, post-intervention, and 6 months post-intervention. Primary outcomes include child weight, diet quality, and neck circumference.

**Discussion:**

This study will be the first to our knowledge to use multiple innovative methods simultaneously including ecological momentary assessment, intervention, video feedback and home visiting with CHWs within the novel intervention context of family meals to evaluate which combination of intervention components are most effective in improving child cardiovascular health. The *Family Matters* intervention has high potential public health impact as it aims to change clinical practice by creating a new model of care for child cardiovascular health in primary care.

**Trial Registration:**

This trial is registered in clinicaltrials.gov (Trial ID: NCT02669797). Date recorded 5/02/22.

## Background

Cardiovascular disease (CVD) is a highly prevalent public health problem [1,2]. CVD is the leading cause of death for one in four adults in the US and affects over 30% of minoritized populations [1]. While CVD peaks in middle age, risk factors begin in childhood and may provide a critical window for intervening to mitigate risk [3]. Children ages 7-10 are at a key age when precursors of CVD begin to be observed, but before the manifestation of disease such as high blood pressure, body mass index (BMI), cholesterol [3,4] and less healthful dietary intake, fewer hours of physical activity, and more sedentary behaviors [5]. To date, there has been low to moderate success with lifestyle behavior interventions for children at risk for CVD and the persistent disparities across race/ethnicity calls for a new and innovative way to intervene [6]. Prior research has identified evidence-based intervention targets and strategies that when combined may provide an innovative approach for improving child cardiovascular health (CVH).

Prior interventions to increase child CVH have not been anchored around a specific family context/routine such as family meals. Instead, interventions often take a “kitchen sink approach” targeting multiple home environment factors (e.g., eating, physical activity, sedentary behavior, parenting) across multiple contexts (i.e., home, school, daycare). These interventions have had limited success [18]. Family meals are unique in that they create a nexus where multiple parenting and familial behaviors related to childhood obesity occur simultaneously (e.g., parent feeding practices, interpersonal behaviors, availability of healthy foods, portion size, modeling healthy eating) and can be intervened on–which rarely occurs in any other context. Furthermore, intervening on one specific context/routine (i.e., family meals) may also seem more doable to parents [19].

Second, some observational studies have shown the need to examine **family meal “quality”** (i.e., dietary intake, interpersonal atmosphere), in addition to family meal quantity, to better understand key protective factors of family meals [7,12,20]. Specifically, prior studies have shown associations between interpersonal interactions (e.g., non-controlling food parenting practices, positive communication/connection) during meals and better diet quality of foods served at family meals (e.g., fruits/vegetables, whole grains), lower child weight status, and higher child diet quality [20,21]. The few existing RCTs examining family meal frequency and child CVD risk found that solely increasing the frequency of family meals was not associated with lower weight status in children [17]. Thus, interventions targeting both family meal quality and quantity will have a higher likelihood of improving child CVH.

Additionally, studies have identified barriers to carrying out family meal routines such as busy schedules, parental stress, lack of food prep/cooking skills, and child behaviors (e.g., picky eating) [22,23]. Research by our team showed that parents experiencing high **stress** levels earlier in the day, were less likely to have family meals, served less healthy foods at mealtimes, and were more likely to engage in controlling feeding practices later the same day [24,25]. Interventions including family meal quantity and quality, as well as strategies to reduce barriers (e.g., stress) to carrying out family meals are needed.

Third, research shows that providing **immediate feedback on behavior** (i.e., ecological momentary intervention (EMI), video feedback) within a specific context (e.g., family meals) results in more behavior change over time [26], compared to solely utilizing parent education [18]. These findings suggest that teaching parents what to do is not enough, rather watching one’s own behavior(s) and receiving feedback that reinforces positive behaviors or prompts different behaviors is necessary. Meta-analyses show that **video feedback** in parenting interventions is feasible, has low participant burden, results in moderate to large effects on parenting behaviors, and results in sustainable behavior change [27].

**Ecological momentary intervention (EMI),** or mobile health (mHealth), uses smartphones to send text messages to participants to intervene on behaviors in real-time as they unfold, moment-by-moment, over time and across contexts [28]. For example, a participant responds to a text earlier in the day regarding their stress level and source(s) of stress (e.g., too many things to do) then, an EMI message is sent providing suggestions to support them in making a healthful choice for family meals in the face of stress (e.g., tip for making a quick pasta meal more healthful by adding a vegetable stir in) [29,30]. EMI studies from other fields have shown significant improvement in targeted behaviors (e.g., medication compliance, smoking cessation) [31,32], high feasibility [32], validity and reliability [33,34], few logistical problems [26], and low burden [35].

Fourth, interventions utilizing **community health workers** (CHWs) who can meet participants “where they are at,” both with regard to readiness for change and in their own environment (i.e., **home visiting**) are associated with better outcomes [36]. CHWs link care across clinic and home contexts and have high success with addressing obesity [36], diabetes [36] and other chronic conditions [36]. In addition, given the rise of **virtual technology** during the COVID-19 pandemic, the need to test virtual versus in-person measurement and delivery of home visiting interventions in a rigorous RCT is key to confirm the benefits of these approaches [37].

**Figure 1:**
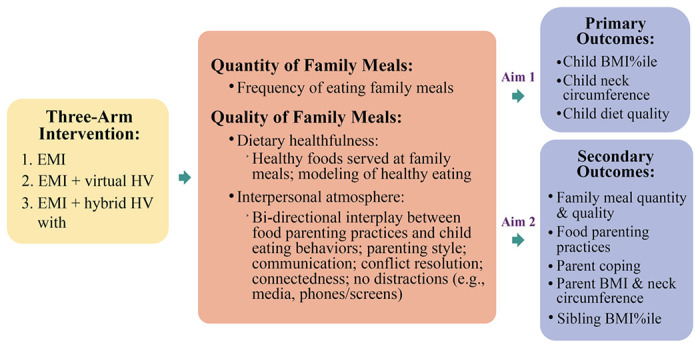
Family Matters Intervention Study.

The **main aim** of the ***Family Matters Intervention*** is to target a well-documented family context associated with child CVH (i.e., family meals) using innovative real-time methods (i.e., EMI, video feedback) with CHWs in both virtual and in-person delivery modes to increase child CVH using a three-arm RCT (see Figure 1). The three Arms include: EMI (Arm 1); EMI+Virtual Home Visiting (HV) with CHW+Video Feedback (Arm 2); and EMI+Hybrid HV with CHW+Video Feedback (Arm 3). Our **overall hypothesis** is that increasing both the quantity and quality of family meals will improve child CVH. Our **main study hypotheses** include (see Figure 1):

- Hypothesis 1: BMI percentile (%ile) and neck circumference will decrease and diet quality will increase in children in Arm 3 compared to children in Arms 1 or 2.
- Hypothesis 2: Family meal quantity and quality will increase, controlling food parenting practices (e.g., restriction) will decrease, and parent coping skills will increase in parents in Arm 3 compared to parents in Arms 1 or 2.
- Hypothesis 3: BMI %ile will decrease in siblings in Arm 3 compared to siblings in Arms 1 or 2.

### Theoretical Framework

Family Systems Theory (FST) [38] guides the current study. According to FST, the family environment is the most proximal influence on child CVH [39,40]. FST suggests that intervening on individual-level behavior (e.g., dietary intake) has limited success unless the family-level behavior sustaining or overriding the individual-level behavior (e.g., fruits/vegetables served at family meals, food parenting practices) changes too [39,41]. FST also suggests that healthful behaviors learned in one family context (e.g., family dinner meal) will generalize to other family contexts (e.g., breakfast, lunch, snacks) [41,42]. Thus, in the current study it is expected that positive parenting practices learned in the family meal context will generalize to other eating occasions and contribute to child CVH overall. Also, including multiple family members (e.g., parents, grandparents, siblings) in the intervention increases the likelihood of sustainable family-level change [7,20].

## Methods

**Figure 2:**
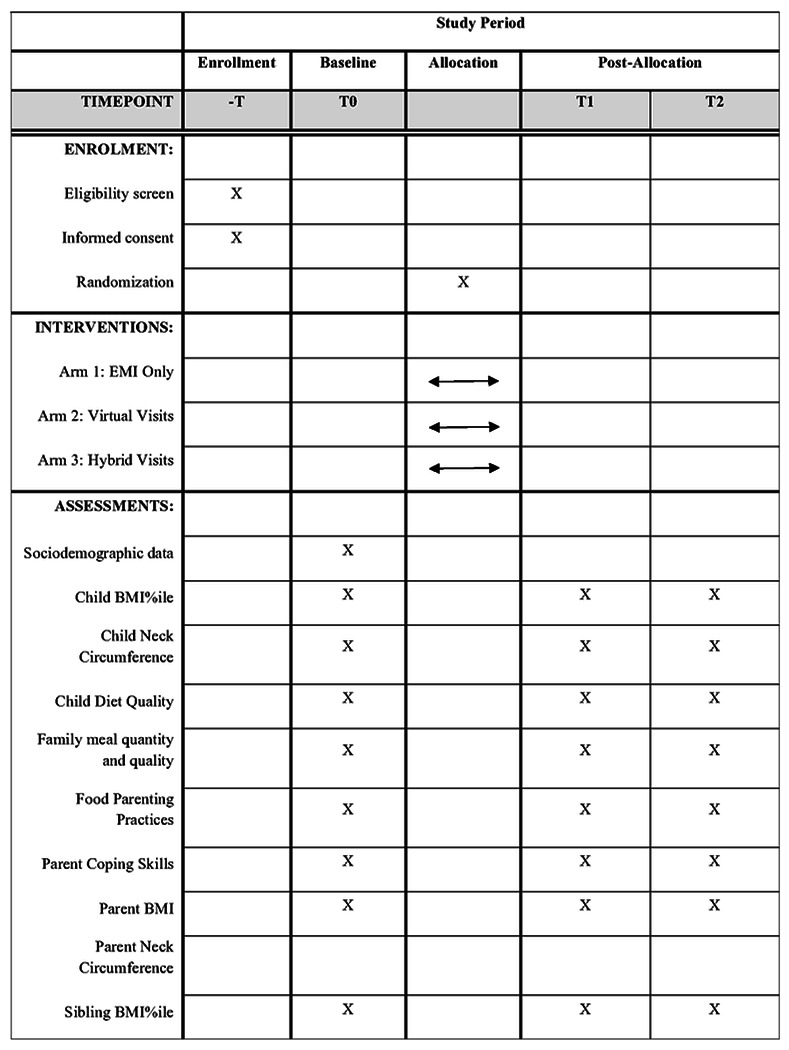
The SPIRIT diagram

This protocol has been written following the guidelines of the Standard Protocol Items Recommendations for Interventional Trials (SPIRIT) checklist (Additional file 1). A SPIRIT figure is also provided below to demonstrate the flow of the study (see Figure 2).

### Study Design

**Figure 3:**
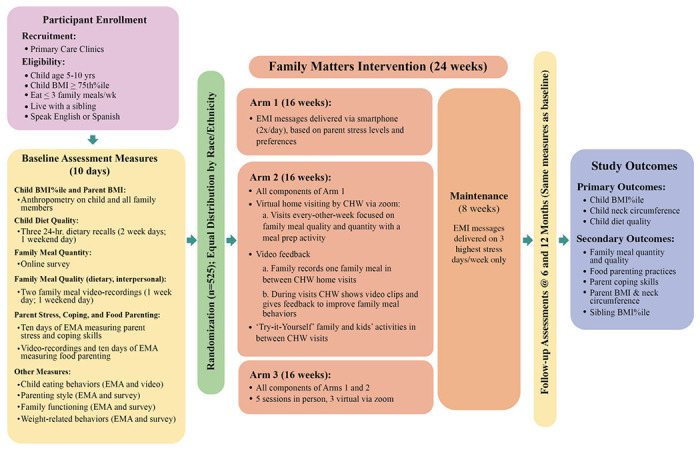
The Family Matters Intervention Flowchart.

The *Family Matters* intervention is a single site RCT with child as the unit of randomization and analysis (see Figure 3). The study is funded by the National Institutes of Health (HL151978) and is registered at clinicaltrial.gov (Trial ID: NCT02669797; May 2, 2022). This RCT lasts 12 months for each family, with a four month active intervention phase, a two month maintenance phase, and data collection at baseline, 6 months (i.e., post-intervention), and 12 months (i.e., 6 months post-intervention). All study materials are created in both English and Spanish.

### Study Recruitment

Children (n=525) and their families are recruited via family medicine and pediatric primary care clinics in Minneapolis and St. Paul, MN. Recruitment is ongoing for 42 months. Eligible children receive a letter inviting participation. Parents then fill out a REDCap survey assessing eligibility criteria.

### Inclusion Criteria:

- Children ages 5-10, their primary caregiver (e.g., parent, grandparent, aunt) and at least one sibling.
- Children at high risk for CVD, defined as BMI ≥75th percentile [43].
- Children from African American/Black, Asian, Hispanic, Native American, or White households who speak Spanish or English.
- Children who consume ≤3 family meals per week [12].

### Exclusion Criteria:

- Children with medically necessary dietary restrictions (e.g., feeding tubes) or who are developmentally unable to participate (e.g., non-verbal).
- Non-custodial parent who lives with the child <50% of the time.
- Children participating in a weight management study.

### Study Arms and Randomization

Families are randomized into one of three intervention Arms: (1) EMI; (2) EMI+Virtual HV with a CHW+Video Feedback; and (3) EMI+Hybrid HV with a CHW+Video Feedback. All Arms receive 16 weeks (4 months) of EMI stress reduction and family meal tip messages via smartphones. Arms 2 and 3 additionally receive eight home visits by CHWs focused on family meal quantity and quality, a meal preparation activity, and video feedback on their family meal behaviors/patterns every-other-week for 16 weeks. Arm 2 receives all of these components virtually and Arm 3 receives these components half in person and half virtual (hybrid). In between weeks, families in Arms 2 and 3 complete a Try-it-Yourself activity to apply the new skills/behaviors they have been taught. All Arms receive an 8-week (2 months) maintenance phase allowing for progressively less support so they can increase self-efficacy and sustainability of behavior change.

Once participants complete their baseline data collection visit, they are randomized into one of three study Arms (n=175 per Arm). If households have multiple eligible children, one child is randomly selected to minimize bias that could affect generalizability due to parent selection. Randomization is stratified by five racial/ethnic groups (African American, Hispanic, Native American, Asian American, White; n≈105 per race group). Block randomization schedules were produced in PASS 2021 (Kaysville, Utah) to account for the racial/ethnic stratification. Schedules are maintained by the biostatistician to keep team members blinded.

### Procedures and Data Collection

#### Virtual Data Collection.

Once child eligibility is confirmed, baseline data collection occurs via a virtual zoom visit including: guided anthropometry [43] and neck circumference measurements, a child 24hr. dietary recall, registration for one-week of Ecological Momentary Assessment (EMA) on their phone [44], and training on video recording of family meals. Following the virtual visit, a 14-day observational period ensues including a parent online survey, two additional 24-hr dietary recalls, ten days of EMA measuring parent stress and parenting practices, and a 2-day video-recorded family meal observation period (1 weeknight, 1 weekend night) measuring family meal quality (i.e., dietary, interpersonal) [45]. Virtual data collection occurs at baseline, 6 months (post-intervention), and 12 months (6-month post-intervention). Primary and secondary outcome measures are described in Table 1 and are collected at all three data collection time points in all Arms. Virtual protocols are based on ours [37] and other’s [7,37] prior studies. Data collection tools and databases (i.e., REDCap) include features to support HIPAA compliance and allows for data checks to ensure data quality during data entry. Access to data collection tools and databases including REDCap and Box will be strictly limited and regulated through personal user profiles. Both of these platforms are password protected and all data will be regularly backed up into a password-protected database.

#### Measures.

This study has three primary child outcomes: BMI%ile [46], neck circumference [47], and diet quality [48,49]. Secondary outcomes include family meal quantity, meal dietary quality [21], meal interpersonal quality [50], parent outcomes: BMI [51], neck circumference [47], food-related parent practices [52], coping skills, sibling BMI%ile, and others [8,13,39,53–60] (see Table 1).

#### Blinding and Investigator Allocation Concealment.

As with most behavioral interventions, it is not possible to double blind this RCT. However, this study incorporates measurement staff and investigator blinding as much as possible to minimize bias. For example, the intervention is administered by CHWs who are not involved with measurement team responsibilities or meetings and measurement team members are blinded to participant study Arm assignment and are not involved with intervention team responsibilities or meetings. The biostatistician is the only completely unblinded member of the research team and will be overseeing data management and analyses throughout the trial and will have restricted access to the final study dataset.

#### Measurement Team Training and Supervision.

Measurement team members are trained, engage in role-plays, conduct mock visits, and are closely supervised by the measurement team director according to best practice [7,63]. Table 2 describes these processes in depth. All practice, certification, and data collection visits are video recorded to allow for thorough supervision of visits where both the measurement team member and their supervisor gives feedback.

Measurement team members are also trained on the Iowa Family Interaction Rating Scale (IFIRS) for video coding of family meals and the Nutrition Data System for Research *(NDS-R)* for dietary recalls [61]. Staff only code families in which they did not participate in the measurement visit [7,20,21]. Practice videos are used until coders reach 95% inter-rater reliability and 100% after consensus meetings; 25% of videos are double coded and checked at a 1:5 ratio to ensure high inter-rater reliability and fidelity to protocols. For *NDS-R*, quality assurance is conducted on 100% of recalls [64].

#### Retention Plan.

To minimize attrition in all study Arms, the following retention strategies are used, based on our successful prior studies with >95% retention rate and best practice [63,65]: (1) gather extensive participant contact information (e.g., phone numbers, email addresses, home/work addresses, emergency contacts); (2) tailor preferred forms of contact to participants (e.g., texts, phone, email); (3) utilize primary care electronic medical record (EMR) databases for updated contact information; (4) send tracking postcards during important cultural celebrations (e.g., Hispanic Heritage Month, Native American Heritage Day); and (5) use ongoing tracking databases (e.g., LexisNexis, White Pages). Additionally, at 9 months families are sent a small gift (e.g., reusable grocery bag with the *Family Matters* logo) and a short survey asking them to update their contact information.

#### Ethical Considerations.

The University of Minnesota’s Institutional Review Board (IRB) Human Subjects Committee approved all protocols used in the study. Prior to enrollment into the study, participants are provided with detailed information about the study by our research team via consent and assent forms including study aims and detailed procedures. Participants are informed that their participation is voluntary and that they have the right to withdraw from the study without any consequences at any point. They will be assured of anonymity in participation and confidentially of any data they provide throughout the study, through the use of study IDs and the storage of sensitive information in secure online platforms (i.e., REDCap and Box). Participants can be enrolled into the study only after they have provided written consent and assent forms to our research team.

#### Regulatory Oversight/Monitoring.

All study modifications will be communicated with and regulated by the IRB. Even though the study is expected to pose minimal risk, the Data Safety Monitoring Board (DSMB), in collaboration with the study investigators will closely monitor recruitment, process evaluation, and retention activities. The DSMB will meet yearly with the study investigators and staff, or more often as needed, for oversight of the study. Any adverse events will be reported to the NHLBI and the IRB at the University of Minnesota. This trial is also registered in the OnCore clinical trial management system and is audited by the Medical School at the University of Minnesota.

## INTERVENTION

The *Family Matters* three-arm intervention, known as the *Family Matters Program* to our study families, components and dose are described below.

### Study Arm #1: EMI

Parents randomized to study Arm 1 receive EMI text messages twice a day for 16 weeks via their smartphone. A study smartphone is provided for use if needed.

#### EMI.

Our prior research showed parental stress early in the day was associated with more controlling food parenting practices and serving more unhealthful foods (e.g., fast food) at dinner the same night [66]. Therefore, in all Arms, parents receive EMI text messages to their phones that include two parts. First, the parent is sent a text message with a survey link, between 11am-2pm to report their stress level (i.e., scale of 0-10) and sources of stress (e.g., child demands, busy at home/work, social media). Second, a text message is sent back to the participant from a bank of tips (approximately 50 tips per source of stress) for the particular source of stress they reported. This tailored tip is intended to help them cope with the reported stress and increase the likelihood that they will still carry out a family meal the same evening in the face of stress [66]. After the tip is sent, parents are also asked to report whether or not the tip was helpful, which then adjusts their individual EMI algorithm so there is an increased likelihood of them receiving more or less of these types of tips. If parents report no stress, they receive a tip to facilitate having a family meal (e.g., recipe ideas, meal prep tips, mealtime conversation starters).

### Study Arm #2: EMI+Virtual Home Visiting (HV) with CHW+Video Feedback

Parents randomized to study Arm 2 receive all elements of study Arm 1, in addition to home visiting by a CHW. Visits by CHWs are virtual via zoom and occur every-other-week (8 total) simultaneously with the 16 weeks of EMI. In between CHW home visits, families complete “Try-it-Yourself” activities (8 total) to reinforce new behaviors and meal preparation skills (e.g., batch cooking recipe, shopping scavenger hunt, stress reduction coping skills).

#### Home Visiting.

CHWs carry out the 60-90-minute HVs using Motivational Interviewing (MI) [67,68] and psychoeducation [35]. The visits focus on family meal quantity and quality factors [7,20,21] known to be associated with child CVH. Family members are taught specific skills through didactic and interactive session activities (e.g., AHA Slides, Figma games) to improve family meal processes and behaviors. Session content and activities are described in Table 3. A SMART goal (i.e., specific, measurable, achievable, relevant and time-bound) [69] is set at the end of each session related to the content delivered in the home visit and their video feedback.

#### Video Feedback.

Every other week, starting during home visit three, families video-record and upload one family meal via their smartphone (6 total meals). CHWs watch videos in between home visits to identify specific clips to show family members at the next visit that highlight both strengths and growth areas regarding interpersonal interactions and dietary patterns. During HVs, CHWs engage family members in a conversation–using MI skills [67] where both the CHW and family members identify positive behaviors seen in the videos and areas for growth, based on session content that families have been learning.

#### “Try-it-Yourself” Activities.

Families are given food-related (e.g., recipes, meal planning strategies) and interpersonal (e.g., food prep with kids, family meal communication game, stress reduction) activities to try out in between visits to increase their self-efficacy in preparing family meals on their own and reinforce messages they are taught during HVs. The study child and all siblings in the home are also given an activity book with games that reinforce session content.

### Study Arm #3: EMI+Hybrid HV with CHW+Video Feedback

Parents randomized to study Arm 3 receive all elements of study Arm 2, but they are delivered hybrid. Specifically, CHWs meet in-person with families every other HV and then virtually via zoom on the other weeks. Families also engage in two cooking demonstration activities with the CHW during in-person HVs to reinforce messages taught, share easy recipes (e.g., batch cooking, one ingredient for multiple meals), and teach food prep skills to increase family’s self-efficacy for having family meals. This Arm is important to examine whether relationship building and creating an atmosphere conducive to health behavior change requires an in-person component. This Arm is also critical to examine COVID’s impact on moving research to virtual modes.

### Maintenance Phase

After completing four months of the intervention, all study Arms transition to a two-month maintenance phase, based on best practice [70]. For all study Arms, EMI family meal tips are reduced to the three days per week that parents reported their highest stress levels during the 16-week active intervention phase. Stress profiles corresponding to the high risk stress days are created for each participant to maximize intervention uptake and subsequent sustainability [24,25].

#### Community Health Workers (CHWs) Training and Supervision.

Interventionists are racially/ethnically diverse CHWs, with half being Spanish speaking. CHWs are trained/certified in MI [67], SMART goals [69], the intervention content for eight HV sessions, video feedback skills [7,27], and HV protocols. The CHW supervision process provides multiple levels of supervision throughout training and intervention delivery (see Table 2). Co-investigators who provide supervision and the intervention director (MA) are trained in MI and are licensed mental health clinicians (JB, TM) or registered dietitians (KL, DNS). All role-plays, certification, and family intervention visits in all study Arms are video-recorded. Both the CHW and supervisor watch and give feedback on the video-recordings, which allows for thorough feedback. Just as families receive video feedback on their recorded family meals from the CHWs as part of the intervention, the CHWs are given feedback as well, thus creating a parallel process that models to the CHW how to give feedback that is collaborative, focusing on both strengths and areas of growth, with their own intervention families.

### Process Evaluation

A robust feasibility and process evaluation protocol was designed for this intervention (see Table 4), to ensure feasibility, generalizability, and dissemination into primary care and other health care settings [70,71].

## STATISTICAL ANALYSIS PLAN

### Overview.

This study is powered for three pairs of tests [72] to evaluate intervention effectiveness: (a) Arm 2 vs. 1, (b) Arm 3 vs. 1, and (c) Arm 2 vs. 3 over three time points. Multi-level, general linear mixed models (MLMM) with a clinic random intercept that nests participants within clinics to address any clinic differences in the recruitment populations, with participant random slopes for time to examine intervention treatment effects, and conditional fixed effects regression models (within-child analytic contrast against baseline), are the primary analytical models for all study hypotheses. Participants’ randomized condition will be examined irrespective of adherence to the study protocol in accordance with an intent-to-treat (ITT) analysis. After the intervention has been fully administered, data will be assessed for balance across arms, outliers, missingness, and other modeling assumptions. Although randomization is expected to produce balance on measured and unmeasured characteristics, variables will be considered for inclusion as controls in adjusted analyses to reduce test statistic variance [73]. We expect little missing data based on our prior work, but if needed, we will employ methods recommended for clinical trials to minimize analytical assumptions required when missing data are present (e.g., follow up all randomized participants prior to unblinding [74], evaluating if results from the primary analysis differ when sensitivity analyses are performed [75]). Reasons for participant withdrawal and non-adherence will be analyzed and reported in the final ITT analysis [76].

### Sample Size and Power Computations.

Study design features were accounted for in powering the study that required increases in sample size to minimize an inflated experiment-wise error rate (EER) due to three pairwise tests between each treatment arm for three primary outcomes (i.e., BMI%ile, neck circumference, and the Healthy Eating Index (HEI)). Accounting for these nine tests, sample size was determined using a conservative two-sided critical value of *z*=2.77 (*P*=0.006) to achieve experiment-wise Type I error of 0.05. Our power calculations were based on prior studies showing that a decline of two BMI%ile points was a clinically meaningful difference in children with overweight/obesity [46]. BMI%ile is a continuous outcome with a variance of 18.8. Eighteen-month follow up data with a comparable cohort provided intraclass correlation coefficient estimates to inform sample size determination (BMI%ile ICC ). At 80% power and multiple-outcome and pairwise testing corrected EER of 0.05, with a sample size of 525, we will be able to detect a minimum average difference in BMI%ile as small as 1.67 (or 0.38 SD) with 15% attrition. This magnitude translates to approximately a 2.8lb difference in a six year-old boy who is 3.8 feet tall and 45 lbs, or approximately a 7.5 ounce per month change in weight by the post-intervention endpoint.

#### Aim 1 (Primary Outcomes): Examine intervention effects on markers of child CVH including BMI%ile, diet quality, neck circumference.

Treatment condition mean differences on the three primary outcomes will be examined at the post-intervention primary endpoint (6 months after baseline). Sample size determination allows for primary outcome standardized effect size assessment of all three outcomes of at least 0.38, which is a small-to-moderate minimum detectable effect.

#### Aim 2 (Secondary Outcomes): Examine intervention effects on family, parental, and sibling factors.

Family meal quantity and quality, food parenting practices and stress, and sibling BMI %ile outcomes are powered at similar levels as in Aim 1 with the ability to detect standardized effect sizes as small as 0.38.

#### Sub-group Exploratory Analyses:

Analyses exploring whether interaction effects depend on participant sex, race/ethnicity, and seasonality will also be conducted. These post-hoc analyses will examine whether the intervention has synergistic effects in specific populations or during different seasonal contexts. Post-hoc analyses will be conducted to explore the interaction of child/parent sex and baseline weight status on intervention treatment effects to determine whether the intervention is particularly effective in certain subpopulations.

#### Other Exploratory Hypotheses:

A model incorporating an interaction effect of treatment arm crossed with the change in family meal quality and quantity between observation periods will be used to evaluate if increases (or decreases) in the quality and quantity of family meals correspond with synergistically favorable (or unfavorable) child outcomes. This analysis will inform whether intervention effects depend on participants’ changes in family meal quality and quantity. This analysis is powered to detect a between-within intervention slope difference over the 6-month intervention period of as little as 9.6 ounces per month, depending on whether participants had high or low change in the moderating variables. Seasonality robustness checks will also be performed to evaluate whether results differ substantively for participants who received the intensive intervention during the summer months.

## Discussion

The *Family Matters* intervention has high potential public health impact as it aims to change clinical practice by creating a new model of care for child CVH in primary care. Research in this field is needed given the low to moderate success of lifestyle behavior interventions for children at risk for CVD and the persistent high prevalence of disparities across race/ethnicity groups. The state-of-the-art measures being used including EMA, EMI, and video feedback combined with the novel intervention context of family meals and CHWs as interventionists will greatly advance the field. In addition, the three-arm study design will allow for testing which combinations of intervention components are most effective in improving child CVH by race/ethnicity as well as whether a virtual or hybrid Arm is more effective. Dependent on study findings, this intervention will be disseminated to other primary care settings.

## Figures and Tables

**Table 1:** Family Matters Intervention Primary and Secondary Outcome Measures and EMI Survey Questions Used in Intervention.

Primary Outcome Measures	
**Child BMI%ile**	Child BMI percentiles (%ile) was used as a primary outcome based on prior evidence that a change in BMI%ile was associated with lower risk of hypertension, dyslipidemia, hypertriglyceridemia, and HDL (risk factors for metabolic syndrome and cardiovascular disease) in children with overweight or obesity [46]. **Procedure:** Objectively measured child height and weight are collected via a virtual visit over Zoom. Families are sent a digital scale and measuring tape. Families watch a short training video on how to take heights and weights. After the video, trained and certified research staff guide parents/guardians on taking the child’s height and weight using standardized protocols [43]. To ensure reliability, all measures are taken twice: height measurements need to be within 0.5 cm of each other, and weight measurements need to be within 1.0 lbs of each other. **Measure**: BMI%iles are calculated using Centers for Disease Control and Prevention (CDC) sex-specific BMI-for-age growth charts [43]. BMI%iles were chosen given the issue of BMI z-scores being a less sensitive measure when children are >95^th^ BMI%tile [46].
**Child Neck Circumference**	Neck circumference (NC) has been shown to be highly associated with cardiovascular disease in children [47] and adults [47], and is strongly correlated with waist circumference [47]. NC is often chosen over other measures (e.g., waist circumference) as it is not impacted by respiratory movement or postprandial abdominal distension, is non-invasive, does not require clothing removal, and is easy to collect [47] **Procedure**: Families are sent a 24” measuring tape prior to their virtual visit to collect NC. Families watch a short training video on how to take NC. After the video, trained and certified research staff guide parents/guardians on taking the child’s NC using standardized protocols. **Measure:** Locate the prominence on the neck (i.e, Adam’s apple), and wrapping the measuring tape around the child’s neck directly under this prominence. The research staff confirm the child is looking straight ahead and that the measuring tape is straight. To ensure reliability, NC measurements are taken twice and measurements need to be within 0.5 cm of each other.
**Child Diet Quality**	Child diet quality was used as a primary outcome given its link to child risk for heart disease and obesity in adulthood. **Procedure:** Three 24-hour dietary recalls (2 weekdays, 1 weekend day) [48] are conducted using the *Nutrition Data System for Research (NDS-R)* [61] regarding the child’s dietary intake. Following best practice guidelines [61], recalls for children <6 years old are done with only the parent. For children >6 years old, recalls are done with both the child and parent; the parent is the main reporter for children 6-8 years old, and the child is the main reporter for children 9+ years old [48]. **Measure:** The first recall is conducted during the virtual visit, and the 2^nd^ and 3^rd^ recalls are scheduled and conducted virtually during the 2-week period following the virtual visit. To increase accuracy of reporting, families are sent a Food Amounts Booklet to measure amounts, school lunch menus are utilized, and parents/children are encouraged to complete a food diary prior to the scheduled recall. An overall *Healthy Eating Index* (*HE*I) [49] score that measures overall diet quality/healthfulness will be calculated for analyses using data from 24-hour dietary recalls.
Secondary Outcome Measures	
**Family-Level Outcomes**	
**Family Meal Quantity**	Family meal quantity was measured as frequency of meals to match prior validated and reliable measures. **Procedure:** Self-report online survey. **Measure:** Parents are asked to report the number of people living in their home. They are then asked to say Yes/No to the following question for each day of the past week: “In the past 7 days, did you have a family dinner meal where at least [# of people in home – 1] – [# of people in home] people were sitting and eating at a table? The online survey is designed to tailor the days of the past week depending on the day the parent is taking the survey. For example, if the parent is taking a survey on a Tuesday and has 5 people in the home, they will be asked about frequency of family dinner meals where at least 4-5 people are present for: Last Tuesday, Last Wednesday, Last Thursday, Last Friday, Saturday, Sunday, and Yesterday.
**Family Meal Quality**	Family meal quality **consists of both dietary quality and interpersonal quality**. These two factors will be measured via video-recorded (via Zoom) family meals as well as ecological momentary assessment (EMA). **Procedures:** · **Video recorded family meals:** Following their virtual visit, families are asked to record two family dinner meals (1 weekday, 1 weekend day) over the next 2 weeks where the majority of the family is present. Our prior research showed that video-recording two family meals, including one week day and one weekend day, was adequate for measuring dietary healthfulness and interpersonal atmosphere at family meals [7,45]. Families are sent a HIPAA-compliant Zoom link where they are able to log on when their meal begins; the link is set up so that recording starts immediately and the video is automatically uploaded to the cloud. At the start of the video, families are instructed to introduce everyone present at the meal and to say what is being served at the meal. · **Ecological Momentary Assessment:** At the virtual visit, staff members register parents for EMA [44], which includes asking about wake and dinner times on weekdays and weekend days. Parents are randomly texted three signal-contingent surveys during the day (determined by parent’s wake time) as well as a meal survey, which is scheduled to be sent a minimum of an hour after the parent reports usually having dinner. Parents use their own smartphone in order to receive texts and access surveys. Parents have 1 hour to finish the first three signal-contingent surveys and 4 hours to finish the meal survey sent at the end of the day. Following the virtual visit, parents have 14 days to finish 10 “complete” days of EMA; a complete day includes at least 2/3 signal-contingent surveys as well as the meal survey. If parents are able to consecutively finish 7 complete days, they earn a $25 bonus. · During the meal survey, parents are asked whether they had a family dinner meal. If they did, they are asked to complete a meal screener. This includes: 1) Listing all the food served at the dinner (e.g., tacos, tortilla chips, refried beans, fruit salad); 2) For each food served, asking about the components of each food, including: Fruits, Vegetables, Dairy, Meat Protein, Other Non-Meat Protein (e.g., tofu), Whole grains, Refined grains, Snack Foods, and Desserts. If a participant selected Vegetables, Dairy, Meat Protein, Snack Foods, Desserts, and/or Sauces, they were asked to categorize the food even further. For example, if Vegetables was selected, the participant was asked if the food contained Dark Green Vegetables (e.g., kale, romaine lettuce, broccoli), Other Vegetables (e.g., corn, tomatoes, peas, potatoes), Fried Vegetables (e.g., French fries), and/or Pickles or olives. · Parents were also asked to report all of the drinks served at dinner, including: Water, Skim/low-fat milk, 2% or whole milk, Non-dairy drinks (e.g., soy milk), Tea, Coffee, Sports drinks, Fruit drinks (e.g., SunnyD), Regular pop/soda, Diet pop/soda, 100% fruit juice, No drinks were served, or None of the above. **Measures:** · **Meal Dietary Quality**. An adapted Healthfulness of Meal (HOM) index [21] is used to measure the dietary quality of foods served at family meals via the EMA meal screener. The HOM index is a quantitative coding system used in prior studies [21]. It was developed based on the Healthy Eating Index (HEI)-2015, which includes 13 components that are either adequacy components (food groups/subgroups that are encouraged) or moderation components (food groups where lower intake is encouraged.) Like the HEI-2015, points are given for the presence of some food components (e.g., Dark Green Vegetables), and the absence of other food components (e.g., Desserts). Higher scores represent a meal with higher dietary quality. · **Meal Interpersonal Quality.** The Iowa Family Interaction Ratina Scales (IFIRS] [50] will be used to measure the interpersonal interactions that occur at the two video-recorded family meals. The IFIRS is a quantitative direct observational coding system that measures behavioral and interpersonal interactions (e.g., dyadic, family level) [50]. IFIRS scales have been used with diverse families and have high validity (r=.77-.86) [50]. Example measures include: parent feeding practices (i.e., restriction, pressure-to-eat), parenting style (i.e., authoritative, authoritarian, permissive, neglectful), and family functioning (e.g., communication, conflict). Higher scores (range 1-9) represent healthy interpersonal dynamics.
**Parent-Level Outcomes**	
**Parent BMI**	**Procedure:** Parent height and weight are taken during the virtual visit using the same procedures as child height and weight. **Measure:** BMI values are computed according to the following formula: weight (kg)/height (meters)^2^ and cut offs for weight status are calculated using CDC guidelines [51].
**Parent Neck Circumference**	**Procedure**: Parent NC is taken during the virtual visit using the same procedures as child NC. **Measure**: The same measure as child NC is used.
**Food Parenting Practices**	**Procedure:** Food parenting practices are assessed via an on-line survey completed by the parent after the virtual visit as well as through the EMA meal survey. **Measure:** Parents are asked about a broad range of food parenting practices [52], which measure structure, autonomy support, coercive control, and indulgent feeding practices [52]. In the on-line survey, parents are asked about the frequency in which they engage in these food parenting practices; in EMA, they are asked (Yes/No) if they engaged in the food parenting practice at the meal that evening.
**Parent Coping Skills**	**Procedure:** Self-report on all four EMA surveys (three signal-contingent and the meal survey). **Measure:** Parents are asked to report on their current stress level (How would you rate your level of stress right now?), coping ability (How would you rate your ability to manage stress right now?), and current sources of stress (Childcare, Job dissatisfaction, Body image/weight concerns, Too many things to do).
**Sibling-Level Outcomes**	
**Sibling BMI%ile**	**Procedure:** Sibling height and weight are taken during the virtual visit using the same procedures as child height and weight. **Measure:** Sibling BMI%iles are calculated using the same procedures as child BMI%ile.
**Other Secondary Outcomes and Sub-analysis Variables**	
	**Procedure:** Other sociodemographic and home environment variables will be measured via validated self-report online measures. **Measures:** Socioeconomic status [53], race/ethnicity [8], sex and gender [54], education [55], acculturation [56], food security [57], food preparation [59], food purchasing behaviors [58,59], media use at mealtimes [13], work/family balance [62], household chaos [39], weight talk [55], and psychosocial factors [60].
**Ecological Momentary Intervention (EMI) Survey Items and Tips Used During the Intervention**	
**Stress Level, Source of Stress, and Helpful/Unhelpful EMI Survey Items**	During the active intervention phase (16 weeks), participants receive a daily text message on their phones with a link to a survey inquiring about: (1) Their level of stress (rating 0-10); (2) Ability to manage stress (rating 0-10); (3) Source of stress–19 potential categories of momentary stressors including (select all that apply): *1.* *Conflicts or arguments* *2.* *Demands from family* *3.* *Traffic/transportation problems* *4.* *New/current events* *5.* *Feeling conflicted over what to do* *6.* *Money for the things you need* *7.* *Too many things to do* *8.* *Job dissatisfaction* *9.* *Upcoming family events* *10.* *Unexpected change in plans* *11.* *Childcare* *12.* *Body image/weight concerns* *13.* *Fatigue* *14.* *Health issues (you own or others’)* *15.* *Interaction on social media* *16.* *Not sure what to serve for dinner* *17.* *Concerns about my child (e.g., wellbeing, behavior problems)* *18.* *Concerns about COVID-19* *19.* *No stress (general tips about family meals)* (4) Primary source of stress (select one option from previous list of sources); and (5) Whether or not the tip they received the day before was helpful. During the maintenance phase (8 weeks), participants receive the same survey 3x/week on the 3 days they reported highest stress levels.
**EMI Tips sent to Participants via Text Message during the intervention**	Tips are sent as text messages to participants based on their reported primary source of stress. A minimum of 50 tips are included in each of the 19 source of stress categories. Tips include affirmations, helpful links/apps, general tips for having family meals including recipes, and games to play at mealtime.

**Table 2:** Training, Certification, Recertification, and Ongoing Supervision for Measurement Team Members and Intervention Community Health Workers.

**Measurement Team Data Collectors (DC) Supervision**		
**Initial Training and Certification**		
Measurement team members go through a 4-6 week training including role-playing and a mock visit with a non-study family to learn how to administer the study measures. Certification is achieved when 95% inter-rater reliability is met for all visit components (i.e., anthropometry, dietary recalls, video-recordings, EMA, online surveys).		
**Ongoing Supervision**		
After training, practice role-plays, and certification, the first 10 virtual data collection visits are reviewed in detail by a supervisor (i.e., measurement team director or co-investigator) with the data collector who conducted the visit. Measurement team members continue to receive feedback on a randomly selected recorded virtual data collection visit by a supervisor on a weekly basis at either an individual supervision session or a group measurement team meeting.		
**Weekly Individual Supervision**	**Weekly Group Supervision**	
**Support for Logistical Issues with Data Collection and Virtual Visits:** Includes troubleshooting issues with recruiting families, scheduling visits, documenting details using RedCap, and other virtual data collection visit delivery logistics/study updates.	**Video Review of Virtual Data Collection Visits:** Includes DCs and their supervisor bringing top strength and growth areas (*with video time stamps*) regarding fidelity to study measures and visit content from previously selected video to team meetings to review/discuss. DCs provide self-feedback verbally first, followed by supervisor feedback.	
**Video Review of Virtual Data Collection Visits:** Includes DC self-feedback and supervisor feedback on strengths and areas of growth in delivering virtual data collection visits regarding fidelity to measures and visit content for one video selected randomly by supervisor. Written feedback given and recorded via RedCap.	**Recertification on Study Measures:** Includes group discussion/review of study protocols; recruitment calls, practice/role-play of virtual visit protocols with other DCs; administration of knowledge quizzes. Successful completion of recertification on study measures documented via RedCap and via video-recordings of virtual visits with participants.	
**Recertification**		
Recertification of measurement team members on all study data collection protocols and measures are conducted every quarter throughout the intervention.		
**Intervention Community Health Workers (CHWs) Supervision**		
**Initial Training and Certification**		
When onboarding, CHWs have 6-8 weeks of training focused on program session content (8 total sessions), foundation training (e.g., motivational interviewing (MI), SMART goal setting, family systems theory, trauma-informed care, food parenting practices, nutrition 101, video feedback) and additional training components (e.g., REDCap, zoom, scheduling visits). They also have the following practice and supervision sessions in preparation for certification and conducting in-home visits:		
**Weekly Q&A Sessions**	**Weekly Group Supervision**	**Weekly Peer Supervision**
Conducted 2-3 times/week. Includes discussion of training materials and logistical components with supervisors.	Conducted twice/week. Includes: - practice/role-plays, mocK visits and certification sessions (with non-study families); attended by supervisors and CHWs and recorded via zoom. - Feedback on video-recorded practice sessions; evaluated by CHW and supervisor. Written feedback given and recorded via RedCap and zoom video. - Evaluation and discussion of recorded family meals (non-study family meals). Written feedback given and recorded via RedCap. Verbal feedback given by CHW and their supervisor and video-recorded via zoom.	Conducted once/week. Includes one-on-one role-play amongst CHWs to practice delivering content for the 8 intervention sessions with a focus on MI skills, SMART goal setting and session content. Recorded via zoom video.
**Ongoing Supervision**		
Once CHWs complete the training/certification period, they continue to receive ongoing supervision and feedback throughout the intervention as follows:		
**Weekly Individual Supervision**	**Weekly Group Supervision**	**Weekly Peer Supervision**
Conducted once/week. **Video Review of Intervention Session:** the first 10 delivered sessions are reviewed in detail by a supervisor with the CHW who conducted the session. Thereafter, 1-2 delivered sessions are selected at random for supervisor and CHW evaluation. The assessment includes CHW self-feedback and supervisor feedback on strengths and areas for growth in delivering intervention sessions with regard to MI skills, SMART goal setting, and session content. Written feedback given and recorded via RedCap and zoom video.	Conducted 1-2 times/week. **Case Consultation Presentation by CHW:** troubleshoot challenges and discuss successes with participant families with supervisors and other CHWs. Written feedback given and recorded via RedCap. Verbal feedback given by CHW and their supervisor and is video-recorded via zoom.	Conducted once/week. **Intervention Delivery Peer Supervision:** Includes one-on-one discussion amongst CHWs about session delivery i.e., successes/challenges/ ideas for improvement and lessons learned. Recorded via zoom video.
**Family Meal Video Feedback for Families:** Includes CHW and supervisor identifying patterns in intervention family’s video recorded family meals and practicing giving feedback with supervisor before delivering the feedback to families. Written feedback given and recorded via RedCap and zoom video.		
**Support with Logistical Issues:** Includes troubleshooting issues with scheduling visits, documenting details using REDCap, using interactive games during session delivery and other intervention delivery logistics.		
**Recertification**		
CHWs are recertified every quarter of the study. Supervisors and CHWs both evaluate two randomly selected intervention delivery videos and asses for fidelity to MI, delivery of session content, and logistical components. Written feedback is given and recorded via RedCap. Verbal feedback is given by CHW and their supervisor and is video-recorded via zoom.		

**Table 3:** Intervention Session Titles, Content, and Interactive Activities for the *Family Matters* Program.

Session Title	Session Content	Session Interactive Activities
Session 1: The Importance of Regular Family Meals	· 3 ingredients of a successful family meal: (1) Have them, (2) Have good communication/interactions, (3) Have healthy food · Health and psychosocial benefits of family meals · Family meal as a ritual · Family meals are doable and benefit all family members	· *Family Matters* Storybook^a^ demonstrating concepts taught during session 1 · Figma^b^ digital flip card game about broad scoping benefits of family meals · Figma^b^ digital flip card myth busting game demonstrating family meals are doable · SMART goal setting focused on ingredient #1: having a family meal
Session 2: Making Family Mealtimes Fun and Healthy	· 3-step communication process (Talk, Listen, and Compromise TLC) to communication and conflict resolution will promote a good family meal emotional environment · Interpersonal connection at family meals and limiting distractions at family meals (e.g., screens) · *Family Matters* mealtime questions card game · Create-Your-Plate with all components of a healthy and balanced meal: grains, proteins, fruits and veggies	· *Family Matters* Storybook^a^ demonstrating concepts taught during session 2 · Google slides^c^ “You’re the Expert” scenarios: ‘What would you do if…?’ game with scenarios: how to use TLC to resolve common family challenges · ‘Let’s Eat Healthy’^d^ digital sort it game: Create-Your-Plate with 4 main categories: whole grains, protein, vegetables, and fruits · SMART goal setting focused on ingredient #2: have good communication and interactions: focus on using TLC
Session 3: Modeling and Food Parenting Practices	· Parents can influence their child’s and family’s eating behaviors: modeling · Division of responsibility “whose job is it?”; food parenting practices and child eating behaviors · Three contexts where food parenting occurs: at mealtime, at home, and outside the home	· High 5 bingo board game: video feedback (for family recorded meal) · *Family Matters* Storybook^a^ demonstrating concepts taught during session 3 · Modeling video:^e^: kids are like sponges · Aha slides^f^: Whose job is it and food parenting contexts: tips from Healthy Eating Research for promoting health eating behaviors in children^g^ · Phrases that help and hinder^h^: how to eat with pick eating behaviors · SMART goal setting focused on ingredient #2: have good communication and interactions: focus on whose job is it and food parenting contexts
Session 4: Meal Planning I for Successful Family Meals: Preparation	· Meal planning strategies and tricks · Power-Up-Your-Plate by adding on or swapping foods that can make your meal healthier · Nutrition Facts Label and ingredients list	· High 5 bingo board game: video feedback (for family recorded meal) · *Family Matters* Storybook^a^ demonstrating concepts taught during session 4 · Meal panning tips and tricks: batch cooking video/ in-person demonstration (arm 3) · Google slides^c^ & coloring sheets for Power-Up-Your-Plate activity: add ons and swaps to create a healthier plate · Nutrition Facts Label digital Interactive sheet^i^ · Aha slides^f^: Nutrition Facts Labels quiz with common/ frequently consumed items (e.g., breakfast cereals) · SMART goal setting focused on ingredient #3: have healthy food: choose a batch cooking recipe to try out
Session 5: Stress Reduction and Barriers to Family Meals	· Stress is normal for both adults and children · There is both chronic and momentary stressors · 4-step process to stress reduction: (1) Name it, (2) Normalize it, (3) Take deep breaths, (4). Find a solution to cope · How to have family meals in the face of stress	· High 5 bingo board game: video feedback (for family recorded meal) · *Family Matters* Storybook^a^ demonstrating concepts taught during session 5 · Figma^b^: chronic vs momentary stress digital worksheet · Canva^j^ stress relief for adults and kids: apps and exercises · Google slides^c^ “You’re the Expert” scenarios: ‘What would you do if…?’ scenarios game: focus on using 4-step process for stress reduction to handle common stressful family situations/challenges · SMART goal setting focused on ingredient #2: have good communication and interactions: focus on using 4-step process for stress reduction; using apps/exercise to reduce stress (coping tools)
Session 6: Meal Planning II for Successful Family Meals: Meal Planning, Shopping, and Involving Kids	· Meal planning should make your life easier, not more stressful · Two-step process for successful meal planning: (1) · Plan for meals ahead of time and (2) Use tricks to facilitate meal planning · Meal planning can be helpful in creating grocery lists and budgeting · Involve your kids in grocery shopping and cooking as developmental appropriate	· High 5 bingo board game: video feedback (for family recorded meal) · *Family Matters* Storybook^a^ demonstrating concepts taught during session 6 · Figma^b^: digital flip card game with tips and tricks to facilitate meal planning · One-ingredient, three recipe cooking demonstration video/ in-person demo (arm 3) · Figma^b^ digital flip card game: kids in the kitchen: match appropriate child age and cooking/cleaning tasks in the kitchen · Food for thought video^k^: summary of main program concepts thus far · SMART goal setting focused on ingredient #3: have healthy food: choose a one-ingredient, 3 meals cooking recipe to try out
Session 7: Snacking and Beverages	· Provide healthy snacks and beverages to your children and create healthy snacking routines · What is my child really asking for?: How to redirect requests for snacks when appropriate · Make water your number 1 choice of beverage	· High 5 bingo board game: video feedback (for family recorded meal) · *Family Matters* Storybook^a^ demonstrating concepts taught during session 7 · Figma^b^ digital flip card activity ‘Did you know game…? About snacking and beverages · Google slides^c^ and live demonstration sugar shocker activity · Aha slides^f^:’What’s really in your snacks? For commonly consumed snack foods (e.g., graham crackers) · Google slides^c^ “You’re the Expert” scenarios: ‘What is your child really asking for…? Scenarios game- focus on snacking behaviors · SMART goal setting focused on ingredient #3: have healthy food: focus on snacks and water intake
Session 8: You are the Family Meal Expert: Celebration!	· You are equipped with the tools and knowledge you need to do this on your own · Barriers happen; your family barriers can be reduced (i.e., identify them and make a plan to mitigate) to ensure future success · Celebrate and Try-It-Forever!!	· High 5 bingo board game: video feedback (for family recorded meal) · *Family Matters* Storybook^a^ demonstrating concepts taught during session 8 · Aha slides^f^: ‘Ask the Expert’ scenarios game: discussion of common scenarios and potential solutions from concepts taught throughout the program · SMART goal setting focused on 3 ingredients of a successful family meal: “try-it-forever”

a Jerica Berge, Katie Loth, and Tai Mendenhall. The Family Matters Storybook, December 2022. Flipbooks https://printing.umn.edu/tools/flipbooks.html

b Figma: the collaborative interface design tool https://www.figma.com.

c Google Slides https://google.com/slides/about/

d Dairy Council of California, Let’s Eat Healthy https://www.healthyeating.org/products-and-activities/games-activities/myplate

e First5LA, Children Are Like Sponges https://www.youtube.com/watch?v=JgBRp13KVBg

f **AhaSlides** https://ahaslides.com

g Healthy Eating Research, Ages 2-8 Feeding Recommendations https://healthyeatingresearch.org/tips-for-families/ages-2-8-feeding-recommendations/

h Phrases that HELP and Hinder https://myplate-prod.azureedge.us/sites/default/files/2020-12/PhrasesThatHelpAndHinder.pdf

i U.S. Food and Drug Administration, Interactive Nutrition Facts Label https://www.accessdata.fda.gov/scripts/InteractiveNutritionFactsLabel/

j Canva Free Design Tool: Presentations, Video, Social Media https://www.canva.com/

k pbs, Eat Grow Thrive | Tips to a Healthy Family for Parents https://www.pbs.org/video/five-fun-tips-to-a-healthy-happy-family-for-parents-tvrsn8/

**Table 4:** Process Evaluation Plan based on the National Institutes of Health Treatment Fidelity Framework [70].

Goal	Execution Strategies
**Treatment Design**	
Ensure the same treatment dose is delivered to each participant within a particular Arm for each of the 3 study Arms	· Ensure fixed number of intervention sessions (8 total), length of sessions (60-90 min), frequency of sessions (biweekly), and duration of intervention protocol (4 months active intervention phase and 2 months maintenance phase) · Ensure fixed amount of information is delivered for each group through scripted intervention protocol (i.e., intervention session scripts) · Video-record all intervention sessions · Evaluate intervention fidelity via coding session videos using a validated system *Motivational Interviewing Treatment Integrity Manual 4.2* (MITI) [71] and provide feedback to CHW interventionists · Document and track all visit and communication details (e.g., REDCap)
Ensure same treatment content (i.e., study learnings, tools and skills provided) is delivered within a particular Arm for all 3 study Arms	· Use of motivational interviewing (MI) techniques and SMART-goal setting during every session · Content for all 8 sessions is scripted and requires certification and recertification by Community Health Worker (CHW) interventionists throughout the intervention · Set goals with a focus on target behaviors related to main intervention learnings (3 ingredients of a successful family meal: (1) Have them; (2) Have good communication and interactions; and (3) Have healthy food
**CHW Interventionist Training**	
Standardize training: ensure that training is conducted similarly for different CHW interventionists	· Use standardized video recorded training materials · Use the same instructors (supervisors) for all CHW interventionists · Conduct the same training content and duration · Conduct the same number of Q&A sessions, role-play/practice sessions, and logistical support sessions · Video-record all role play/practice and Q&A sessions for supervision and to serve as a reference for future training · Have CHW interventionists train together · Apply the same certification procedures to CHW interventionists
Ensure CHW interventionist skill acquisition: include well-defined performance criteria	· Evaluate intervention implementation via video recordings · Code and score CHW interventionist adherence to protocols in session delivery (MI- specific evaluation adapted from the MITI coding manual and checklists for session content) · Conduct regular debriefings and problem solving sessions · Certify CHW interventionists before intervention session delivery
Minimize drift in CHW interventionists skills: measure skill acquisition throughout and post-training	· Conduct ongoing (weekly) role-play/practice and supervision sessions with CHW interventionists in individual and group supervision formats · Supervisor and Self-Evaluation of CHW interventionists’ sessions delivered to families · Recertify CHW interventionists quarterly during the 5-year intervention
**Treatment Delivery**	
Ensure adherence to intervention protocol with regard to content and treatment dose	· Video-record intervention sessions, supervisor and self-evaluation e and review with the CHW interventionists (video-feedback) · Code delivered sessions for fidelity to MI using the MITI coding manual and provide feedback to CHW interventionists
Reduce differences in intervention delivery	· Use scripted intervention protocol and session materials · Have supervisors evaluate video-recorded delivered sessions using a MI-specific guide and pre-developed session specific checklists · Code delivered sessions for fidelity to MI using the MITI coding manual
Control for CHW interventionists differences (i.e., assess nonspecific treatment effects)	· Assess participant’s relationship with the CHW interventionists via mid and end of intervention surveys and provide feedback to CHW interventionists · Have supervisors evaluate video-recorded delivered sessions using MITI coding system · Have CHW interventionists work with all intervention Arms
**Treatment Receipt**	
Ensure participant comprehension and understanding of information provided in the intervention	· Have CHW interventionists ask questions/ discuss materials with participants (during *follow-up and understand new concepts* segments in every intervention session) · Use MI techniques that prompt CHW interventionists to paraphrase/ summarize content · Assess comprehension by playing games to apply intervention concepts and messages · Set SMART- goals (at the end of each intervention session) that target main intervention concepts and learnings and follow-up on achieving or revising set goals as needed · Evaluate recorded family meals for application of intervention concepts and provide video-feedback to families to reinforce positive behaviors and address areas for growth
Ensure participant’s ability to perform behavioral skills	· Have CHW interventionists ask questions/ discuss materials with participants in every intervention session · Assess participant completion of assigned activities · Set SMART goals with participants and trouble shoot issues in accomplishing goals set · Evaluate recorded family meals for application of intervention concepts and provide video-feedback to families · Collect and analyze outcome data
**Treatment Enactment**	
Ensure participant’s use of skills in appropriate life settings	· Evaluate recorded family meals for application of intervention messages and provide video-feedback to families · Collect and analyze outcome data at post-intervention and 6-month post-intervention

## Data Availability

The datasets generated from the study will be available from the corresponding author upon reasonable request.

## Abbreviations

CVH: Cardiovascular Health
EMI: Ecological Momentary Intervention
CHW: Community Health Worker
HV: Home Visiting
FST: Family Systems Theory
EMA: Ecological Momentary Assessment
IFIRS: Iowa Family Interaction Rating Scale
NDS-R: Nutrition Data System for Research
EER: Experiment-Wise Error Rate
HEI: Healthy Eating Index
